# ENHANCING MUSCLE FUNCTION IN FRAILTY: FINDINGS FROM A PILOT STUDY ON MAGNETIC MITOHORMESIS THERAPY

**DOI:** 10.1093/geroni/igae098.2758

**Published:** 2024-12-31

**Authors:** Hae Sagong, Pao-Feng Tsai, Chih-Hsuan Wang, Lynn Brown, Jos Edison

**Affiliations:** Auburn University, Auburn, Alabama, United States; Auburn University, Auburn, Alabama, United States; Auburn University, Auburn, Alabama, United States; Auburn University, Auburn, Alabama, United States; Auburn University, Auburn, Alabama, United States

## Abstract

Magnetic Mitohormesis Therapy (MMT) employs low-amplitude pulsing electromagnetic fields (PEMF) and has demonstrated the ability to enhance muscle function and metabolism. Given that muscle weakness is a key contributor to frailty, this study employed a double-blind pilot randomized controlled crossover trial design involving prefrail/frail participants without neurodegenerative diseases, from two distinct types of long-term care facilities. Eight Participants underwent 12 weeks of twice-weekly treatment followed by 12 weeks of sham control, or vice versa, with two groups randomly assigned: Sham-Treatment (S-T group) and Treatment-Sham (T-S group). Treatment was delivered using The QuantumTx BIXEPS Fitness and Wellness device delivered PEMF at 1.5 mT flux densities. FRAIL Scale, SPPB, SF-12v2 Health Survey, SWAY Cognitive Tests, and Body Composition Measurements were assessed at every 6 weeks. Results showed that the S-T group demonstrated improved performance in the Gait Speed test. Their scores from the Gait Speed test were higher at 24 weeks compared to baseline (p=.01), and their overall SPPB scores were higher at 24 weeks compared to baseline (p=.01). Participants noted the study site’s accessibility and described the treatment as quick and straightforward. This study provided preliminary evidence of the benefits of MMT in frail older adults; however, further research with a larger sample size and shorter study duration is needed to lower the dropout rate and mitigate the development of new health issues.

